# Management of Type 2 Diabetes With Insulin Glargine-100 in Iraq in a Real-Life Observation

**DOI:** 10.7759/cureus.31164

**Published:** 2022-11-06

**Authors:** Abbas A Mansour, Abbas Rahmah, Mahmood Khudhair

**Affiliations:** 1 Diabetes and Endocrinology, Faiha Specialized Diabetes, Endocrine and Metabolism Center (FDEMC) University of Basrah, Basrah, IRQ; 2 Department of Internal Medicine, National Diabetes Center, Mustansyriah University, Baghdad, IRQ; 3 Department of Internal Medicine, College of Medicine, Al-Nahrain University, Baghdad, IRQ

**Keywords:** glargine-100, iraq, glycemic parameters, basal insulin, type 2 diabetes mellitus

## Abstract

*Introduction: *Type 2 diabetes mellitus (T2DM) management is challenging in conflict zones, such as Iraq, and insulin might not be readily prescribed or available to patients who need it. This study describes the profile of Iraqi T2DM patients treated with insulin glargine-100 (Glar-100) and their response to the treatment.

*Research design and methods:* This observational, multicenter registry collected data over six months on demographic and medical history, insulin regimen, changes in glycemic parameters, and vital signs in patients with T2DM in Iraq.

*Results:* Patients (N = 245) were 55.2 ± 10.2 years old, with 58.4% females. They had had diabetes for 10 years, with baseline glycated hemoglobin (HbA1c) levels of 10.3% ± 1.6% (89 mmol/mol), diabetes complications, and co-morbidities. Almost all were on oral anti-diabetics before treatment intensification with Glar-100. Over 50% of patients with prior insulin exposure were receiving basal plus or bolus regimens at baseline, versus 6% of insulin-naïve patients, though some required treatment intensification. Most were asked to self-titrate according to fasting plasma glucose (FPG), but self-titration proved challenging. Upon Glar-100 treatment, HbA1c and FPG levels significantly decreased (*P *< 0.001), and 20% of patients (mostly insulin-naïve) achieved HbA1c < 7% (53 mmol/mol). Vital signs improved, while weight changes were modest. Most safety events were mild, and only two patients discontinued Glar-100.

*Conclusion*. Glar-100 safely and effectively decreased FPG and HbA1c levels, achieving HbA1c control in some patients. Glar-100 is a promising T2DM therapeutic option in Iraq.

## Introduction

Type 2 diabetes mellitus (T2DM) is a progressive multi-system disease with variable degrees of deteriorating beta-cell function and insulin resistance, resulting in increased blood glucose levels [1,2] and leading to premature death. In fact, it was estimated that five million diabetic patients died from diabetes in 2015 worldwide [3].

Two out of five patients with T2DM in the Middle East and North Africa region are left undiagnosed, contributing to the global estimates of about 382 million people with diabetes, of which 90% were cases of T2DM in 2015 [4]. Undiagnosed and thus untreated diabetes puts patients at high risk of developing microvascular and macrovascular complications [5,6]; underscoring the urgency of glycemic control. Diabetes-related myocardial infarction, stroke, amputation, and microvascular disease might be delayed or prevented by each 1% decrease in glycated hemoglobin (HbA1c) levels, as reported by the UKPDS study [6].

The prevalence of T2DM in Iraq is estimated between 8.5% and 13.9% [7], contributing to the worldwide increasing trend that projects 366 million people with T2DM in 2030 [8]. A more recent study reported a 19.7% age-adjusted prevalence of diabetes among adults in the city of Basrah, Iraq [9].

In addition, and since the 1991 embargo following the Gulf War, the healthcare system in Iraq has started declining, which was only exacerbated during the 2003 war [10,11]. This situation created barriers to self-care among patients in general and patients with T2DM in particular, given the complicated nature of the disease and the dependence on medical supplies and insulin [10,12]. Although insulin is not required by all patients with T2DM, it might be indispensable to achieve good glycemic control [13], due to a progressive decline in beta-cell function or to the failure of oral anti-diabetes drugs (OADs) [14]. In fact, a recent consensus on the management of T2DM in Iraq advises the use of insulin as a third-line therapeutic, if oral monotherapy and dual therapy fail [15].

Insulin glargine-100 (Glar-100) is a long-acting insulin analog commonly used as a replacement for basal insulin. It is modified to deliver consistent levels of plasma insulin over an extended duration, and it has been shown to lower HbA1c levels and the risk of hypoglycemia [13,16,17]. Despite established therapeutic efficacy, little is known about the treatment of T2DM in Iraq in general and about the use of Glar-100 in particular, especially since insulin might not always be available at the primary care level [18].

This study aimed to describe the profile of patients treated with Glar-100 in terms of age, diabetes duration, HbA1c and FPG levels at baseline, prior anti-diabetes medication, etc. Secondary aims of this study included a description of the insulin titration scheme and change in insulin dosage throughout the study duration, patients achieving HbA1c control after six months of treatment, changes in glycemic parameters and weight at three and six months, in addition to estimating the frequency and severity of adverse events (AEs; notably hypoglycemia). Findings were also compared in terms of prior insulin use (naïve versus previously insulinized populations).

## Materials and methods

Study design and participants

This was a national, multicenter, prospective observational product registry over a six-month follow-up period. This study did not impose any additional procedures, assessments, or changes in the routine management of patients.

Investigators were endocrinologists and internal medicine physicians from Iraq who decided to intensify their patients' treatment with Glar-100 after judging that their previous anti-diabetes therapy had failed.

Eligibility criteria included being older than 18 years, having T2DM, being on oral or injectable anti-diabetes medications other than Glar-100, having uncontrolled HbA1c (≥7% [53 mmol/ml]) in the three months prior to the study, and being treated with Glar-100 independently of study entry. Type I diabetes mellitus, prior treatment with Glar-100 within the year leading up to the study, hypersensitivity to Glar-100, pregnancy, breastfeeding or lack of efficient contraception for women of childbearing age, mental disease, and participation in another clinical study constituted exclusion criteria. All included patients signed informed consent.

This registry was conducted per the principles laid down by the 18th World Medical Assembly (Helsinki, 1964), including all subsequent amendments; it was in line with the International Conference on Harmonization's "Good Clinical Practice" and "Good Epidemiological Practice" guidelines.

The study was approved by the Iraqi Ministry of Health and the Institutional Review Board of the Faiha Specialized Diabetes, Endocrine, and Metabolism Center (FDEMC) of the Basrah Directorate of Health (Reference number 56/35/22).

Data collection and management

Data were collected using paper case report forms (CRF) at baseline, at three months (±one week) and then at six months (± one week) of follow-up. Collected data were checked and verified by study monitors against source documents. During the computerized handling of the data, all errors and inconsistencies were raised and resolved before the database was considered clean and final.

All AEs were collected regardless of their seriousness or relationship to Glar-100. Serious AEs were recorded within 24 hours of awareness. Non-serious AEs were reported within 30 days of awareness. All AEs were reported on the corresponding page(s) of the CRF.

Statistical considerations

Sample Size Determination

With 267 patients, the study would allow the estimation of any mean or average (such as the mean age of patients, the mean duration of diabetes, the mean baseline HbA1c, etc.) within 12% of the value of its standard deviation (SD) using 95% confidence intervals (CI). Also, this sample size would allow the estimation of any prevalence within a maximum margin of error of 6% using 95% CI for proportions. Allowing for a 10% loss to follow-up, the study aimed at recruiting 297 (rounded up to 300) patients.

Analysis of Endpoints

Numerical variables were summarized using means, SD, and range. Categorical variables were summarized using frequency distributions.

Absolute and relative changes in HbA1c levels from baseline to six months were presented as means ± SD and estimated using a 95% Wald CI. Moreover, testing for significant differences in HbA1c between baseline and six months was done using the paired t-test or a Wilcoxon signed rank test, depending on the normality of the data. Based on the HbA1c levels after six months of treatment, participants were categorized into those with controlled (HbA1c < 7% or 53 mmol/mol) or uncontrolled (HbA1c ≥ 7% or 53 mmol/mol) glycemia using a 95% Wald CI. Patients were stratified according to their previous anti-diabetic medications (prior insulin treatment versus insulin-naïve patients). Differences in the main and secondary outcomes were assessed using an independent t-test or a Wilcoxon/Mann-Whitney test, depending on the normality of the numeric variables, and the chi-squared test or the Fisher’s exact test, depending on the expected counts for categorical variables.

Modulation of FPG levels, weight changes, and changes in Glar-100 doses were assessed at three and six months, and significance was evaluated using the paired t-test/Wilcoxon signed rank test. The time to reach glycemic control was estimated from the two visits, and the average Glar-100 dose was computed.

Handling of Missing Data

The last observation carried forward (LOCF) method was used in cases of missing data for numeric variables and the non-responder imputation method for categorical variables. Only the results after imputation were presented. All analysis was performed using IBM SPSS (version 20; Armonk, NY: IBM Corp.), and a two-tailed P-value ≤ 0.05 was deemed significant.

## Results

Description of study participants

A total of 245 patients were recruited from 27 clinics across Iraq. With 16 patients lost to follow-up beyond the baseline visit (missing HbA1c levels at six months), the analysis population consisted of 229 patients. Data from these 229 patients were analyzed for efficiency of treatment and were distributed as follows: 199 (86.9%) were insulin-naïve upon enrolment in the study, while 30 (13.1%) had previously been treated with insulin (with the exception of Glar-100). Table 1 displays the baseline characteristics of study participants.

**Table 1:** Characteristics of study participants at baseline BPM: beats per minute; DPP-IV: dipeptidyl peptidase IV. *Significantly different between insulin-naïve patients and patients who were on insulin prior to the study (P < 0.001). Numerical results are displayed as mean ± SD; categorical results are displayed as n (%).

	N = 245	
Age, in years	55.2 ± 10.2	
Male gender, n (%)	102 (41.6%)	
Clinical profile		
Body mass index, in kg/m^2^	30.2 ± 5.5	
Blood pressure	in mmHg	in kPa
Systolic	138±17.4	18.4
Diastolic	83.8±9.3	11.17
Heart rate, in bpm	80.6 ± 8.9	
Diabetes history		
Duration, in years	9.3 ± 5.9	
Complications, n (%)	N = 143 (58.4%)	
Diabetic neuropathy	114 (79.7%)	
Diabetic retinopathy	37 (25.9%)	
Angina pectoris	26 (18.2%)	
Diabetic nephropathy	19 (13.3%)	
Co-morbidities, n (%)	N = 171 (69.8%)	
Hypertension	148 (86.6%)	
Dyslipidemia	122 (71.4%)	
Oral anti-diabetes medication prior to the study, n (%)*	N = 240 (97.9)	
Biguanides (metformin)	225 (93.8%)	
Sulfonylureas	193 (80.4%)	
DPP-IV inhibitors	62 (25.8%)	

Anti-diabetic therapy at baseline

Baseline Glycemia Data

Baseline FPG and HbA1c levels were recorded for all 245 patients (Table 2). FPG ranged from 95 to 540 mg/dL, with a mean of 223.4 ± 61.9 mg/dL (95% CI, 215.6-231.2). Self-titration aimed at achieving and maintaining target FPG levels, which were set between 100 and 150 mg/dL. Baseline HbA1c levels varied between 7.2% (55 mmol/mol) and 15.5% (146 mmol/mol), with a mean of 10.3% ± 1.6% (95% CI, 10.1-10.6, or 89 mmol/mol). None of the glycemic parameters were significantly different between insulin-naïve and previously insulinized patients at baseline.

**Table 2:** Glar-100 titration scheme FPG: fasting plasma glucose; HbA1c: glycated hemoglobin; IU: international units

	Insulin-naïve patients	Previously insulinized patients	Overall N = 245
Glycemia at baseline			
HbA1c, in %	10.4 ± 1.6	10.2 ± 1.8	10.3 ± 1.6
HbA1c, in mmol/mol	90 mmol/mol	88 mmol/mol	89 mmol/mol
FPG, in mg/dL	224.2 ± 61.7	218.1 ± 63.6	223.4 ± 61.9
Target FPG, in mg/dL	124.7 ± 11.5	121.8 ± 11.9	124.3 ± 11.5
Self-titration scheme	N =112	N =9	N =121
Daily	6 (5.4%)	2 (22.2%)	8 (6.6%)
Every three days	68 (60.7%)	5 (55.6%)	73 (60.3%)
Weekly	38 (33.9%)	2 (22.2%)	40 (33.1%)
Insulin increment, in IU	2.4 ± 1.5	2.4 ± 1.3	2.4 ± 1.5

Insulin treatment regimen at baseline

All patients who attended the baseline visit had been treated with Glar-100 at the sole discretion of their treating physician, independently from the study. Therefore, all 245 patients of the study population were treated with Glar-100 (basal insulin): 187 patients (94.0%) of the insulin-naïve group were treated with Glar-100 alone, 10 (5.0%) were following a basal plus regimen, and two (1.0%) were following a basal-bolus regimen. However, patients who were treated with insulin prior to the initiation of Glar-100 were significantly differently distributed (P < 0.001). Out of the 30 insulinized patients, 14 (46.7%) were switched to Glar-100 alone, 11 (36.7%) were recommended a basal plus regimen, and 5 (16.7%) were recommended a basal-bolus regimen.

Insulin Dose at Baseline

Though the distribution of insulin-naïve and previously insulinized patients across the different insulin regimens was statistically significant, administered doses were not. The Glar-100 dose ranged between 6 and 40 international units (IU), with a mean of 17.2 ± 6.3 IU and a 95% CI of 16.4-18.0 for the whole population (P = 0.128 when comparing the two patient groups). Regarding fast-acting insulin and regardless of prior insulin exposure, the overall dose was 10.5 ± 3.4 IU, and most patients in the basal plus regimen administered their fast-acting insulin at breakfast (71.4%) and less than a quarter of them at lunch (23.8%). The average dose for fast-acting insulin at baseline for patients following the basal-bolus regimen was higher than the dose for basal plus patients, with 12.3 ± 4.6 IU at breakfast, 12.7 ± 5.1 IU at lunch, and 13.1 ± 4.7 IU at dinner. Although the average doses received by insulin-naïve patients were much lower than those received by patients with prior insulin exposure, only seven patients were following a basal-bolus regimen at baseline, and the difference failed to reach statistical significance.

Glar-100 Titration Scheme

A total of 121 patients (60.2%) were instructed to self-titrate their Glar-100 to match their needs and target FPG. Table 2 describes the different self-titration schemes performed at baseline by insulin-naïve and previously insulinized patients. Titration schemes performed as a baseline, as well as HbA1c, baseline, and target FPG levels were not significantly different among the two groups. Insulin incremental units ranged from 1 to 15 for insulin-naïve patients and from 1 to 5 for previously insulinized patients, and there was no significant difference between the two groups.

OAD treatment

A total of 208 (97.7%) insulin-naïve patients were treated with OADs in parallel with insulin therapy, while 28 (87.5%) of the previously insulinized patients received OADs (P < 0.05), making up 236 (96.3%) of the total study population. The difference in OAD prescription across insulin regimens and between insulin-naïve patients and those previously treated with insulin was not significantly different.

Analysis of efficiency of anti-diabetes treatment

FPG Levels Greatly Decreased After Three and Six Months of Treatment With Glar-100

**Figure 1:**
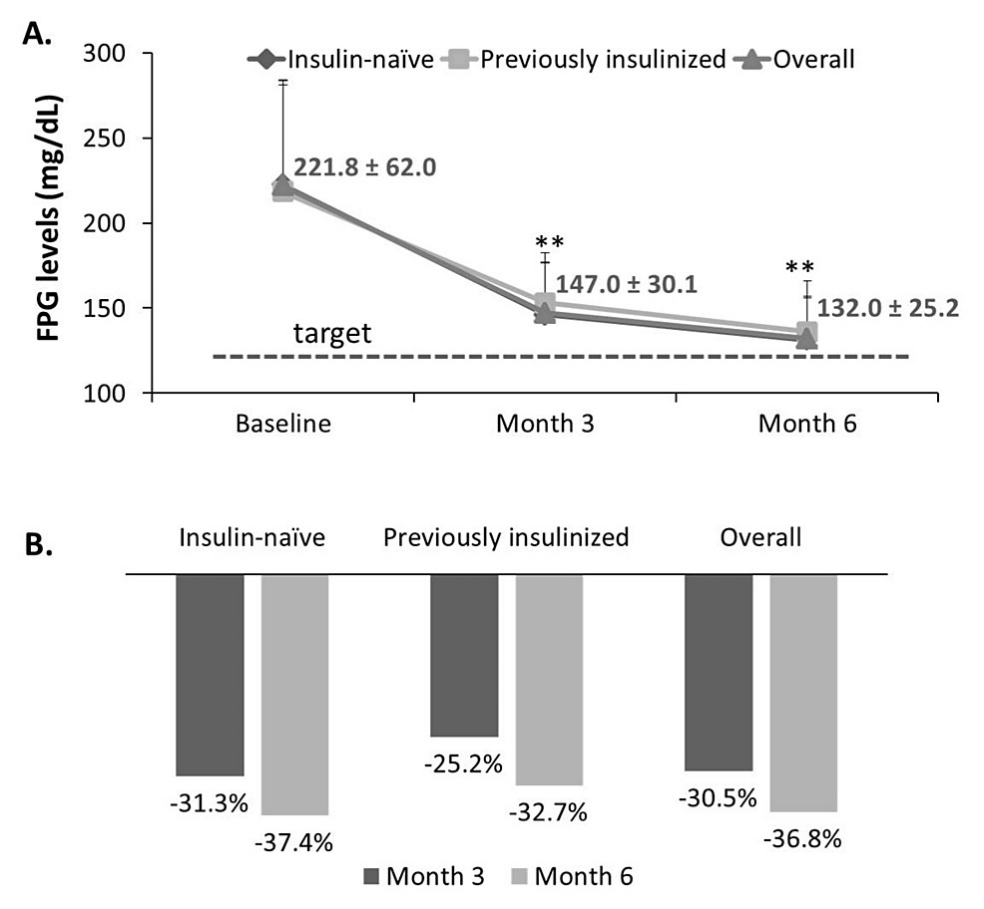
Treatment with Glar-100 was associated with lower FPG levels. FPG levels were recorded at baseline, at month 3 then at month 6 post initiation of Glar-100. Panel A: FPG levels decreased significantly throughout the experimental duration. Data labels correspond to overall FPG levels. Panel B: Bar graph showing the percentage reduction in FPG at month 3 and month 6. FPG: fasting plasma glucose. **P < 0.001 using the Wilcoxon signed-ranks test.

At baseline, the overall FPG ranged between 95 and 540 mg/dL, with a median of 210 and a mean of 221.8 ± 62.0 mg/dL, and decreased to 147.0 ± 30.1 mg/dL (P < 0.001) after three months of treatment, then to 132.0 ± 25.2 mg/dL (P < 0.001) after six months of treatment with Glar-100 (Figure 1A), closer to the set FPG target of 124.3 ± 11.5 mg/dL. Figure 1B shows the reduction percentage in FPG levels across data points. Insulin-naïve patients showed a higher response to Glar-100 (a reduction of 31.3% at month 3 and 37.4% at month 6) than previously insulinized patients (a reduction of 25.2% at month 3 and 32.7% at month 6), but this difference did not reach statistical significance (P = 0.420, using the Mann-Whitney U test).

Glar-100 Successfully Decreased HbA1c Levels

Reductions in FPG also translated into a decrease in HbA1c levels and a higher proportion of patients achieving the HbA1c target of less than 7% (53 mmol/mol). HbA1c levels were significantly decreased in both the insulin-naïve group and the previously insulinized group, with an overall percent reduction of 16.8% after three months of Glar-100 introduction and 23.3% after six months of treatment (P < 0.001) among the 229 patients eligible for analysis.

**Figure 2:**
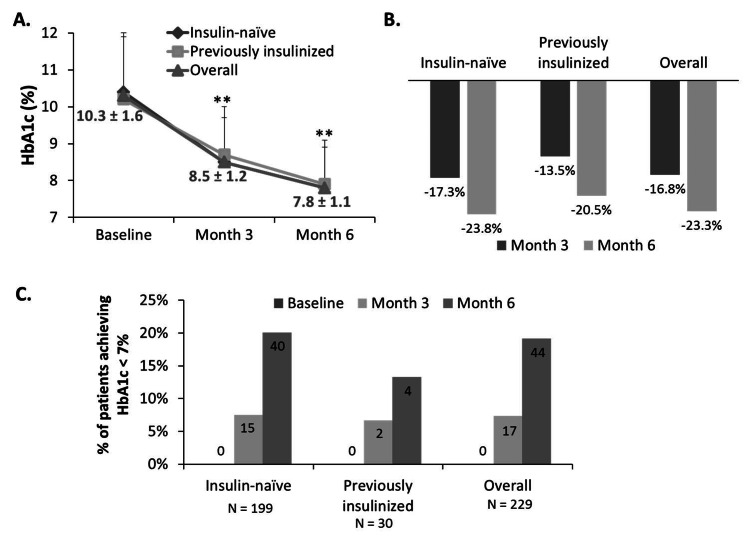
Glar-100 successfully decreased HbA1c levels. HbA1c levels were recorded at baseline, at month 3 and then at month 6 post initiation of Glar-100. Panel A: HbA1c levels decreased significantly throughout the experimental duration. Data labels correspond to overall HbA1c levels. Panel B: Bar graph showing the percent reduction in HbA1c at month 3 and then month 6 from baseline. Panel C: Bar graph showing the numbers (data labels) and percentages of patients achieving HbA1c target < 7% (53 mmol/mol) in the different subgroups and overall, at all data points. HbA1c: glycated hemoglobin. **P < 0.001 using the Wilcoxon signed-ranks test.

Figure 2A displays consistently decreasing levels of HbA1c from baseline to months 3 and 6, overall and in both insulin-naïve and previously insulinized patients. The percent reduction in HbA1c levels is displayed in Figure 2B, showing that HbA1c levels decreased in a time-dependent manner. Reductions of HbA1c levels from baseline to months 3 and 6 reached statistical significance with P < 0.001, while differences between insulin-naïve and previously insulinized groups were statistically insignificant (P = 0.253, using the Mann-Whitney U test). Importantly, despite the modest achievement of HbA1c targets at month 3 (7.4%), a greater proportion of patients (19.2%) reached the HbA1c target in month 6 (Figure 2C).

Changes in insulin regimens and doses throughout the study

**Figure 3:**
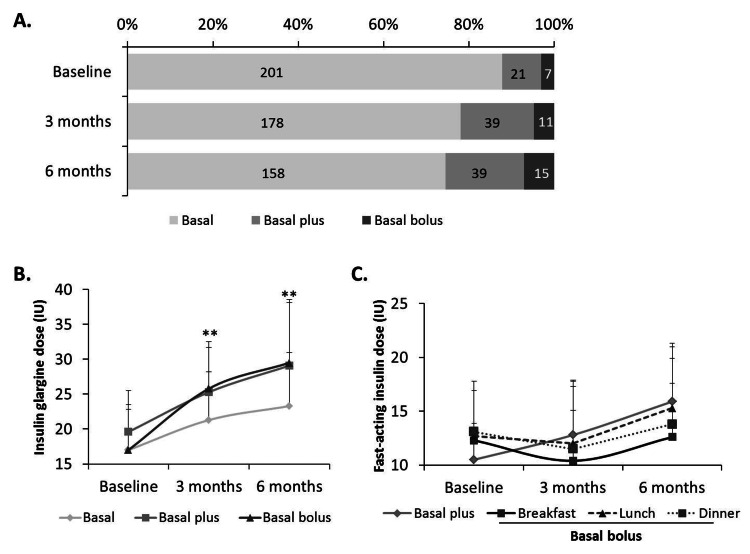
Changes in insulin regimens and insulin dose throughout the visit. Insulin regimens and daily doses were recorded at baseline, month 3 and month 6. Panel A: Proportions (and number) of patients who were treated with Glar-100 in a basal, basal plus or basal bolus regimens at baseline and the changes that occurred at the subsequent study visits. Panel B: Glar-100 doses increased over the study duration. Panel C: Changes in fast-acting insulin over the study duration in patients on basal plus or on basal bolus (at breakfast, lunch and dinner). **P < 0.001.

Throughout the study visits, most patients were on Glar-100 alone, with intensification occurring during the study duration (Figure 3A). In fact, insulin treatment was escalated from baseline to either basal plus or basal-bolus for 29 (12.7%) patients at month 3 and for 9 (4.3%) patients at month 6. The mean daily dose of Glar-100 at baseline was 17.2 ± 6.3 IU, and it was significantly increased to 22.2 ± 7.0 IU after three months (P < 0.001) and to 24.4 ± 8.5 IU after six months (P < 0.001, Figure 3B). Though average doses in the previously insulinized patient group were higher at all three data points, there was no significant difference between insulin-naïve and previously insulinized patients regarding the mean Glar-100 dose at month 6 (P = 0.553). For patients on basal plus, fast-acting insulin doses increased over the study duration, while patients in the basal-bolus group had a decrease in the mean fast-acting insulin doses at the second visit (month 3), to increase again at month 6 (Figure 3C).

Self-titration schemes were also analyzed throughout the study to evaluate target FPG and insulin dose increments, in addition to patients' compliance with their self-titration scheme. At the three-month visit, 23 patients (10.0%) were found to be non-compliant with the self-titration scheme, down to 12 patients (5.7%) at the six-month visit, irrespective of prior experience with insulin. Reasons for non-compliance were mostly a generally poor compliance attitude of the patients (around 61% at month 3 and 58% at month 6), followed by complaints that the titration scheme was not practical (around 13% at month 3 and 25% at month 6). None of the non-compliant patients attributed their related AEs to non-compliance with the self-titration scheme.

Change in body weight

The analysis of body weight across the study data points suggests a rather modest change from baseline to month 3 and then month 6, reflected in a non-significant difference according to the Wilcoxon signed-ranks test.

However, a closer look shows a greater (however modest) reduction in body weight among previously insulinized patients (−1.46% at month 6, reflecting a decrease from 80.9 ± 11.5 kg to 79.4 ± 9.4 kg), compared to insulin-naïve patients (+0.61% at month 6, reflecting an increase from 81.8 ± 15.9 kg to 82.0 ± 15.0 kg). This difference between groups reached statistical significance (P < 0.05), according to the Mann-Whitney U test. Analyzing the BMI of patients in this study shows that previously insulinized patients had a BMI decrease of 1.46% ± 5.6% compared to a 0.60% ± 5.24% increase among insulin-naïve patients (P = 0.044), six months after the study started.

Change in blood pressure and heart rate

At study visits, blood pressure (systolic [SBP] and diastolic [DBP]) measurements were reported. By month 3, SBP had decreased by 6.0 ± 15.1 mmHg (or about 0.8 kPa; equivalent to a 3.5% ± 10.2% decrease, P < 0.001); and this decrease was more pronounced by month 6 (4.9% ± 10.6%; P < 0.001). The same trend was observed for DBP, which went from 83.6 ± 9.1 mmHg (around 11.1 kPa) at baseline to 80.9 ± 6.6 mmHg (10.8 kPa) at month 3, and 80.3 ± 6.4 mmHg (10.7 kPa) at month 6. Though the decrease in DBP was not significant for the 30 patients with prior insulin treatment, the change in SBP across the study visits was significant for the overall population of 229 patients (P < 0.001). Changes in SBP and DBP were not significantly different whether patients were treated with insulin prior to Glar-100 or not. Heart rate was also reported at study visits, and the data reveal that insulin-naïve patients had a drop in their heart rate from 80.9 ± 9.1 beats per minute (bpm) to 78.7 ± 6.4 bpm at month 3 and 78.9 ± 6.7 bpm at month 6. A decrease in heart rate between baseline and each study visit was significant, with P < 0.001 at month 3 and P < 0.005 at month 6. Patients with prior insulin use had an increased heart rate from 78.9 ± 8.7 bpm at baseline to 81.8 ± 6.9 bpm at month 6, and the difference with insulin-naïve patients was significant (P = 0.037).

Analysis of safety events

Safety data were recorded for all 245 patients who attended the baseline visit. A total of 45 patients (18.4%) experienced 48 AEs. Most AEs were mild (44 AEs out of 48), three were moderate, and one was severe (acute coronary syndrome). In addition to being described as severe, this case of acute coronary syndrome and another case of retinal hemorrhage were considered serious, and they required hospitalization.

As for their relationship to Glar-100, 24 AEs were attributed to the treatment, of which 17 were among the patients newly started on insulin. Of those 24 AEs, weight gain (9 cases) was the most common. In addition, two patients discontinued Glar-100 due to AEs (a case of elevated liver enzymes and another one of central abdominal pain).

The study team followed up on the outcome of the 48 AEs reported during this study. Most were either recovered or recovering (34 cases); four had not recovered by the end of the study, and six outcomes remained unknown.

## Discussion

Although lifestyle modifications remain the cornerstone for T2DM management, a large proportion of patients will require pharmacotherapy, starting with OADs, with the possibility of requiring lifelong daily injectable therapies [15] to prevent or delay diabetes-related complications. Despite the advances in T2DM pharmacotherapy, poor glycemic control is still prevalent and might be due to undertreatment and limited self-care [19,20]. In fact, controlling HbA1c to target levels below 7% (53 mmol/mol) might not be easily achieved [13,19,21]. Many patients will eventually require and benefit from insulin therapy, and basal insulin alone remains the most convenient initial insulin regimen. Glar-100 is a soluble, long-acting insulin analogue that is commonly used as a replacement for basal insulin. It is modified to deliver a consistent level of plasma insulin over an extended duration to lower HbA1c and reduce the risk of hypoglycemia [13,16,17].

This prospective study was designed to identify the profile of Iraqi patients who are treated with Glar-100 by their endocrinologist or internal medicine practitioner in Iraq upon failure of prior insulin-based or oral anti-diabetic therapy.

Patients who participated in this study were adults with an average age of 55 years, no gender predilection, and a BMI in the pre-obesity and obesity ranges according to the World Health Organization classification [22].

Some patients have had T2DM for up to 34 years, with a median of nine years, possibly explaining the high prevalence of diabetes-related complications reported. The average duration of T2DM reported in this study (9.3 ± 5.9 years), the frequency of complications and comorbidities, as well as the elevated BMI range, match recent reports in Lebanon, another Middle Eastern country [23].

A study published in 2020 also described the demographic and clinical profile of a large cohort of T2DM patients in Iraq, treated at the tertiary care level [24]. Obesity, long-term T2DM, and dyslipidemia were found to predict poor glycemic control; which can explain the results reported in the present study. In fact, despite the significant decrease in HbA1c levels over the six-month study duration, glycemic control remains modest in terms of short-term (FPG) and longer-term (HbA1c) glycemic parameters. Noteworthy, all patients had uncontrolled glycemia at study entry, and the introduction of Glar-100 to their T2DM pharmacotherapy did lower HbA1c and FPG levels and yield HbA1c control in a non-negligible proportion of the population. However, other factors might hinder glycemia control, as discussed in the literature [24-26].

Most patients were on OADs, in addition to newly initiated Glar-100, and the most common OADs were metformin, sulfonylureas, and DPP-IV inhibitors, also consistent with recommended practice in Iraq [15]. Over half of the study patients were following a Glar-100 self-titration scheme (daily, every three days, or weekly); and although close to 20% of patients achieved HbA1c control (<7% or 53 mmol/mol), clinical evidence from treat-to-target randomized controlled trials points to better glycemic control after six months of treatment [13,27]. This difference might be due to a relatively low final average dose of daily insulin (24.4 ± 8.5 IU, up from the baseline average of 17.1 ± 6.2 IU) and failure to properly self-titrate Glar-100. Self-titration of insulin doses might indeed be challenging for T2DM patients, especially for subgroups with lower health literacy levels. The 2020 study in Iraq reported that 56% of T2DM patients who participated in it had inadequate health literacy levels [26], possibly contributing to limited compliance with insulin titration schemes. In addition, over 88% of study patients were on Glar-100 (basal alone regimen); which might suggest that a larger proportion of patients could reach glycemic control if prandial insulin is more frequently added to their treatment.

Hypoglycemia represents a major AE experienced by patients with T2DM [28,29]. During the six-month study, neither hypoglycemia nor substantial weight gain was reported during the Glar-100 treatment duration, confirming further the safety of Glar-100 in terms of hypoglycemia and weight change [30].

Limitations

First, a six-month-long observation may be too short to draw any conclusions about the clinical effectiveness of a long-term treatment such as Glar-100. Second, no analysis was performed to describe the therapeutic combinations (OAD + Glar-100 + prandial insulin) that better reduced glycemic parameters (HbA1c and FGP). Third, the sample size of 245 patients may not completely represent the T2DM Iraqi population. These limitations, however, did not compromise the reliability of the reported primary outcomes of this study. In fact, the findings are consistent with a larger retrospective study conducted in Iraq and published in 2020 [24].

## Conclusions

Glar-100 successfully decreased FPG and HbA1c levels in this Iraqi population of patients with T2DM (diagnosed around a decade prior to this study) within six months of Glar-100 initiation. The large majority of patients were on basal insulin (Glar-100 alone), and only a limited proportion of patients required treatment intensification to basal plus or basal-bolus. While all study patients had uncontrolled HbA1c levels at baseline, more patients in the insulin-naïve group achieved the HbA1c target (<7% or 53 mmol/mol) than those in the previously insulinized group. Vital signs (including SBP, DBP, and heart rate) improved over study visits, while weight changes were rather modest and not significant. There was an overall low rate of safety reporting in the present study, which indicates the need to raise awareness among investigators on the importance of collecting and reporting AEs. Importantly, diabetes management needs to be stepped up in Iraq to achieve glycemic control, and Glar-100 seems to constitute a promising therapeutic option for T2DM in Iraq.
